# The Effect of Ball Milling Time on the Isolation of Lignin in the Cell Wall of Different Biomass

**DOI:** 10.3389/fbioe.2021.807625

**Published:** 2021-12-14

**Authors:** Guangrong Yang, Xueying An, Shilong Yang

**Affiliations:** ^1^ College of Furniture and Industrial Design, Nanjing Forestry University, Nanjing, China; ^2^ School of Landscape Architecture, Jiangsu Vocational College of Agriculture and Forestry, Jurong, China; ^3^ State Key Laboratory of Pharmaceutical Biotechnology, Department of Sports Medicine and Adult Reconstructive Surgery, Nanjing Drum Tower Hospital, The Affiliated Hospital of Nanjing University Medical School, Nanjing, China; ^4^ Advanced Analysis and Testing Center, Nanjing Forestry University, Nanjing, China

**Keywords:** biomass, milled wood lignin, β-O-4, molecular weight, functional group

## Abstract

Ball milling technology is the classical technology to isolate representative lignin in the cell wall of biomass for further investigation. In this work, different ball milling times were carried out on hardwood (poplar sawdust), softwood (larch sawdust), and gramineous material (bamboo residues) to understand the optimum condition to isolate the representative milled wood lignin (MWL) in these different biomass species. Results showed that prolonging ball milling time from 3 to 7 h obviously increased the isolation yields of MWL in bamboo residues (from 39.2% to 53.9%) and poplar sawdust (from 15.5% to 35.6%), while only a slight increase was found for the MWL yield of larch sawdust (from 23.4% to 25.8%). Importantly, the lignin substructure of *ß*-O-4 in the MWL samples from different biomasses can be a little degraded with the increasing ball milling time, resulting in the prepared MWL with lower molecular weight and higher content of hydroxyl groups. Based on the isolation yield and structure features, milling time with 3 and 7 h were sufficient to isolate the representative lignin (with yield over 30%) in the cell wall of bamboo residues and poplar sawdust, respectively, while more than 7 h should be carried out to isolate the representative lignin in larch sawdust.

## Introduction

As the depletion of fossil energy and its derived environment issues, a sustainable alternative program is sought urgently. Lignocellulosic biomass is regarded as a clean and renewable resource and shows potential to replace petrochemicals to produce biochemicals, biopolymers, biofuels, etc. (Himmel et al., 2007; Pei et al., 2020; Lin et al., 2021; Zhao et al., 2021a). Hence, developing lignocellulosic biomass to produce green energy and biomaterials is the main concern of scientists in this century. All of lignocellulosic biomass is composed of the cellulose, hemicellulose, and lignin with different proportions (Hu et al., 2021; Zhang et al., 2021; Zhao et al., 2021b). Currently, the application of cellulose and hemicellulose have been much investigated and converted into energy chemicals in industry (Huang et al., 2016a; Chen et al., 2018; Lai et al., 2019; Luo et al., 2021). However, lignin, the major phenolic polymers in biomass, remains underutilized in biorefining, which is required to explore the potential applications in theory (Liu et al., 2021a; Liu et al., 2021b; Huang et al., 2022). Actually, many works have shown that lignin possesses the potential to be further converted into different chemicals and materials, such as phenolic resins, dispersants, binders, carbon fibers, and active substance (Ragauskas et al., 2006; Yang et al., 2007; Jiang et al., 2017a; Zheng et al., 2021a). In addition, lignin is composed of cross-linked phenylpropanoid units, including *p*-hydroxyphenyl (H), guaiacyl (G), and syringyl (S). In the network of lignin biomacromolecule, both of these units are linked by inter-unit linkages of carbon–carbon (β-β, *ß*-5, *ß*-1, and 5–5′) and ether bonds (β-O-4′, *a*-O-4′, and 5-O-4′) (Martín-Sampedro et al., 2019). Therefore, due to the highly variable complex structure of lignin, how to effectively separate lignin from lignocellulosic biomass is the key to efficient utilization of lignin (Yuan et al., 2013).

Now, a variety of extraction methods has been reported to isolate the lignin in biomass. Generally, the extraction methods can be divided according to the used solvent, such as alkaline lignin, lignosulfonate, organic solvent lignin, milled wood lignin (MWL), Klason lignin, and cellulolytic enzyme lignin (Lange et al., 2013; Huang et al., 2017b; Huang et al., 2018; Yun et al., 2021). Different types of lignin show different characterizations of lignin. Even though these methods have been proposed, there are still some issues. For example, the structures of lignin can be severely degraded during alkaline extraction process (alkaline lignin and organic solvent lignin) and condensed during acid hydrolysis process (Klason lignin) (Yuan et al., 2010; Sun et al., 2013; Jiang et al., 2020). For cellulolytic enzyme lignin, a lot of enzymes should be used to degrade the carbohydrate that linked to lignin, and the obtained lignin sample still possesses a high amount of carbohydrate (Guerra et al., 2006). Currently, MWL is considered as the most comprehensive method of keeping native-like lignin structure from biomass (Zhang et al., 2010). The MWL separation method was proposed by Bjorkman in 1956, which uses the solvent of dioxane/water to extract the sufficient milled lignocellulosic biomass (Bjorkman, 1956). It has been reported that the structure of MWL has only minor changes occurring during the extraction process of lignin, which is very fit with the lignin structure in the original biomass. However, the extraction method of MWL also has unexpected problems in practical application of yields of lignin, which is dependent on the ball milling time (Wang et al., 2019; Wang R. et al., 2021). Therefore, this method is only widely used in theoretical research, contributed to provide more accurate theoretical foundations for industrialized applications of lignin. Technologically, ball milling should be carried out to isolate MWL from biomass. However, due to the diversified and complex cell structure of biomass, different ball milling time should be carried out to isolate the representative MWL from different biomass.

Technologically, many advanced technologies have been investigated to characterize the structure of lignin, such as gel permeation chromatography (GPC), Fourier transform infrared spectroscopy (FTIR), nuclear magnetic resonance technology (NMR), etc. (Huang et al., 2017a; Xu et al., 2011; Zhang et al., 2010; Martín-Sampedro et al., 2019). NMR is a state-of-the-art analytical technique that can be applied to analyze the structural features of the lignin samples from different biomasses (Liao et al., 2020). The NMR technology is considered to be non-destructive technology for lignin during analysis process, which is able to detect different nuclei of the different linkages in lignin. Meanwhile, NMR is a structure-sensitive analysis technology, which can get the structural information of lignin in solution phase and solid phase (Liao et al., 2020; Halleraker and Barth., 2020; Zhao et al., 2021a). Hence, the application of NMR technology to analyze the structure of lignin can provide detailed and comprehensive information about them (Luykx et al., 2008). Specifically, the ^1^H NMR spectroscopy can get the hydrogen (proton) signal information of lignin, which can be subdivided into different structural regions (Azhar et al., 2007). The quantitative methods of ^13^C NMR spectroscopy can measure the carbon signals at different categories of C structures in lignin (Capanema et al., 2004). Meanwhile, 2D heteronuclear single quantum coherence (HSQC) NMR technology has also been developed to get the quantitative information of lignin structures, including lignin aliphatic and aromatic areas (Ibrahim et al., 2012; Tang et al., 2021). In addition, ^31^P NMR spectroscopy has also been widely used to quantify the functional groups (hydroxyl and the carboxyl groups) of lignin (Hussin et al., 2014). Overall, with the scientific research of this century focused on the comprehensive utilization of lignin, the NMR is considered to be an important lignin structure analysis technology.

Even the influence of ball milling on the efficiency of MWL isolation and structure has been substantially studied. There is no comparative work showing how the structure of MWL samples from different biomass species changes by ball milling at the same milling time. In this work, different ball milling times of 3–7 h were carried out for different biomass of bamboo residues, poplar sawdust, and larch sawdust. This is aimed to provide an appropriate protocol to obtain the representative MWL for further application. To understand the effect of ball milling time on the structure of prepared MWL samples, the chemical composition, molecular weight, lignin substructure, and content of functional groups were analyzed by high-performance liquid chromatography (HPLC), gel permeation chromatography (GPC), 2D-HSQC nuclear magnetic resonance (NMR), and quantitative ^31^P NMR, respectively.

## Materials and methods

### Materials

The moso bamboo residues, poplar sawdust, and larch sawdust were collected from the furniture process factory in Fujian, China. Before the analysis of the major components in all sawdust, they were ground into particles with the size of 2–5 mm and then extracted by benzene/ethanol (2:1, v/v) for 16 h to remove the solvent extractives. The major components of the bamboo residues, poplar sawdust, and larch sawdust were 43.5%, 46.6%, and 41.8% of glucan, 19.5%, 19.8%, and 21.7% of xylan, and 35.4%, 28.3%, and 29.4% of lignin, respectively.

### Isolation and purification of lignin from different biomasses

The lignins in the biomass of bamboo residues, poplar sawdust, and larch sawdust were isolated according to the standard method proposed by Bjorkman (1956). Specifically, 10 g of wood sawdust particle was loaded in a 100-ml ZrO_2_ bowl with 25 ZrO_2_ balls (ΦA = 1 cm). Then the particle was milled by the planetary ball milling under a rotation speed of 600 rpm for a total effective time of 3, 5, and 7 h. During milling process, a milling time of 10 min and a pause time of 15 min were carried out for the planetary ball milling instrument. After milling, all ball-milled meals were collected to extract the lignin using a mixture of 1,4-dioxane and water (96:4, v/v) with a liquid-to-solid ratio of 20:1 for 24 h. The extraction process was repeated three times with a fresh mixture of 1,4-dioxane and water. After three times of extraction, all filtrates were mixed and evaporated by vacuum rotary evaporation at 45°C to get the crude lignin solid, which was termed as milled wood lignin (MWL). To purify the crude MWL, the solid was dissolved in a mixture of acetic acid and water (90:10, v/v) with a liquid-to-solid ratio of 20:1 (ml:g). Then the dissolved lignin in the mixtures was precipitated by adding distilled water. The precipitated MWL was washed with water several times and freeze dried to get the solid. The dried MWL solid was dissolved in a mixture of dichloroethane and ethanol (2:1, v/v) with a liquid-to-solid ratio of 10:1 and then precipitated by adding ether (10 ml/ml of dichloroethane/ethanol). The precipitated MWL was sequentially washed by ether and petroleum and then air dried to obtain the purified MWL.

### Component analysis of biomasses and prepared lignins

The major components of three sawdust and the purified MWL preparations were measured according to the standard procedure proposed by the National Renewable Energy Laboratory (NREL) (Sluiter et al., 2011). Specifically, 30 mg of extractive-free sawdust particles (20–40 mesh) or MWL powder was hydrolyzed by 0.3 ml of 72% H_2_SO_4_ at 25°C for 1 h. The hydrolyzed substrate by 72% H_2_SO_4_ was diluted to 4% H_2_SO_4_ with distilled water and auto-hydrolyzed at 121°C for 1 h. The sugar contents in the acid–hydrolyzate were analyzed by high-performance liquid chromatography (HPLC, Agilent 1,260), which is equipped with HPX-87H column and refractive index (RI) detector. The acid-soluble lignin in the acid–hydrolyzate was determined by the ultraviolet spectrophotometer at 205 nm. The acid–insoluble lignin was determined by weighting the residual solid (oven dried) in the acid–hydrolyzate. The lignin content in three sawdust and the purified MWL preparations was referred to the total amount of acid–soluble lignin and acid–insoluble lignin.

### Molecular weight analysis of prepared lignins

The molecular weight determination for the prepared lignins was carried out by gel permeation chromatography (GPC). In order to improve the dissolving capacity of lignin in the tetrahydrofuran for GPC analysis, all MWL samples were acetylated according to the work of Pan (2008). For analysis, 2 mg of acetylated lignin was dissolved in 2 ml of tetrahydrofuran and ejected into the GPC system, which is equipped with a PL-gel 10 mm mixed-B 7.5 mm i.d. column and an ultraviolet detector. The analysis was performed at ambient temperature using tetrahydrofuran as the eluent (1 ml/min). Monodisperse polystyrene was used as the standard to calibrate the molecular weight of lignin in the GPC system.

### 2D-HSQC nuclear magnetic resonance analysis of prepared lignins

To get the information of the substructure of lignins from different biomasses under various milling times, 2D-HSQC NMR analysis was carried out using a Bruker AVANCE 600 MHz NMR spectrometer equipped with a 5-mm BBO probe. For analysis, 100 mg of oven-dried lignin was dissolved in 400 μl of DMSO-d_6_ and then transferred into an NMR tube. The acquisition data points for F2 (1H) dimension and F2 (13C) dimension were 1,024 (53 ms) and 256 data points (5.14 ms), respectively. The total delay time and scan times were 1.5 s and 160 for the acquisition process, respectively. The 2D-HSQC NMR spectra were processed using the TopSpin software (4.0.5 version).

### Functional groups analysis of prepared lignins

Quantitative ^31^P NMR was carried out to analyze the functional groups using the NMR spectrometer (the same as 2D-HSQC NMR analysis). For analysis, 0.04 g of oven dried lignin and 0.5 ml of anhydrous pyridine/CDCl_3_ mixture (1.6:1, v/v) were introduced into the NMR tube to dissolve the lignin. Then 0.2 ml of internal standard solution (endo-N-hydroxy-5-norbornene-2, 3-dicarboximide, 9.23 g/L), 0.05 ml of relaxation reagent solution (chromium (III) acetylacetonate, 5.6 g/L) and 0.1 ml of phosphitylating reagent solution (2-chloro-4,4,5,5-tetramethyl-1,2,3-dioxaphospholane) were sequentially added into the NMR tube for analysis. The acquisition parameters for ^31^P NMR analysis were according to the standard protocol in NMR spectrometer.

## Results and discussion

### Effect of different milling times on the isolation yield and chemical composition of lignin from different biomass

As the balling milling is the first step to isolate the MWL sample as the native-like lignin in wood and different pretreated wood, it is important to understand how the milling time affects the isolation yield and structural changes of lignin in different woods. In this work, ball milling with 3, 5, and 7 h were performed on the different biomass (bamboo residues, poplar sawdust, and larch sawdust) to isolate the native-like lignin, which is aimed to provide an appropriate protocol to obtain the representative lignin for further application. In Table 1, it can be seen that the isolation yields of each MWL sample from different biomass were linearly increased with the milling time from 3 to 7 h. These results were in accordance with the work of Capanema et al. (2015), who reported that the MWL yields of birch and maple could be enhanced from 8% to 28% and from 12% to 30% with an increased milling time from 2.5 to 10 h, respectively, while it should be pointed out that there are some differences in the isolation yields of MWL (based on the Klason lignin in biomass) from bamboo residues, poplar sawdust, larch sawdust, which were in the range of 39.2%–53.9%, 15.5%–35.6%, and 23.4%–25.8%, respectively. It is reported that MWL preparation isolated from biomass with a yield over 30% can be regarded as the representative lignin in cell wall (Huang et al., 2016a). Hence, the results showed that milling times with 3 and 7 h were sufficient to isolate the representative lignin in the cell wall of bamboo residues and poplar sawdust, respectively, while, more than 7 h should be carried out to isolate the representative lignin in larch sawdust. This might be due to the more tough cell structure of larch than that of bamboo and poplar.

**TABLE 1:** The isolation yield and composition content of prepared milled wood lignin (MWL) samples from different biomass with different milling times.

Biomass	Milling time (h)	Isolation yield of MWL (%)	Composition content of MWL (%)				
			Xylan	Arabinan	Glucan	Mannan	Lignin
Bamboo residues	3	39.2	4.9	1.1	0.8	/	91.5
	5	45.7	4.9	0.8	0.6	/	92.8
	7	53.9	3.6	0.8	0.6	/	94.6
Poplar sawdust	3	15.5	5.8	0.9	0.2	/	90.5
	5	24.3	6.5	0.7	0.5	/	92.4
	7	35.6	5.6	0.6	0.3	/	92.8
Larch sawdust	3	23.4	/	/	2.8	6.8	90.1
	5	24.8	/	/	1.1	5.6	92.1
	7	25.8	/	/	1.0	5.4	92.9

For the isolated lignin from the cell wall of biomass, the residual carbohydrate in the MWL preparation can affect its dissolving capacity in the DMSO-d_6_ for further analysis by NMR technology. Hence, the composition content of all prepared MWL samples were analyzed and shown in Table 1. From Table 1, it can be seen that both MWL preparations from bamboo residues, poplar sawdust, and larch sawdust contained carbohydrates of 5.0%–6.8%, 6.5%–7.7%, and 6.4%–9.6%, respectively. For the existing carbohydrates in MWL preparations, xylan was the major carbohydrate in MWL preparations from bamboo residues and poplar sawdust, while mannan was the major carbohydrate in MWL preparations from larch sawdust, which was due to their different biomass species. In this work, all the MWL preparations were obtained from the crude lignin, and after tedious purification procedures, there are still some carbohydrates in the MWL preparations. This can be explained by the hemicellulose, which is covalently linked to lignin by phenyl glycoside linkages (PhGlc), benzyl ethers (BE), and -esters (Est), which is termed as lignin–carbohydrate complexes (LCC) (Zheng et al., 2021b; Gu et al., 2021). Technically, the existing LCC in the cell wall of biomass can be degraded during the milling process, while it is hard to remove during the purification process by various solvents, which can be the reason why the prepared MWL samples from different biomass cannot reach to a purity of 100% (Henriksson, 2017). Overall, the obtained MWL preparations from bamboo residues, poplar sawdust, and larch sawdust contained over 90% of lignin.

### Molecular weight analysis of prepared milled wood lignin samples

To verify how the milling time affects the prepared MWL samples in bamboo residues, poplar sawdust, and larch sawdust by different milling times, the weight average molecular weight (M_w_) and number average molecular weight (M_n_) were analyzed and are shown in Table 2. It can be seen that the M_w_ values of the prepared MWL sample bamboo residues, poplar sawdust, and larch sawdust decreased from 8,577 to 5,324, 6,509 to, and 16,296 to 13,345 g/mol, respectively, when the milling increased from 3 to 7 h. These results indicated that the increased milling time can decrease the molecular weight of prepared MWL from biomass. In the work of Guerra et al. (2006) and Wang R. et al. (2021), it was also reported that the intensive milling time can show the performance by decreasing the molecular weight of lignin in the cell wall of different biomass. In addition, Table 2 shows that both of the prepared MWL samples from different milling times possessed PDI values lower than 3, indicating that the isolated MWL samples possessed a relative homogeneousness (Kim et al., 2011; Huang et al., 2016b).

**TABLE 2:** The molecular weight of prepared MWL samples from biomass at different milling times.

Biomass	Milling time (h)	Molecular weight (g/mol)		Polydispersity index (PDI)
		Weight average (M_w_)	Number average (M_n_)	
Bamboo residues	3	8,577	4,754	1.80
	5	5,411	3,456	1.57
	7	5,324	2,816	1.89
Poplar sawdust	3	6,509	3,430	1.90
	5	6,337	3,214	1.97
	7	4,330	2,436	1.78
Larch sawdust	3	16,296	7,230	2.25
	5	14,105	4,888	2.89
	7	13,345	4,848	2.75

### Effect of different milling times on the structure of prepared milled wood lignin samples

**FIGURE 1:**
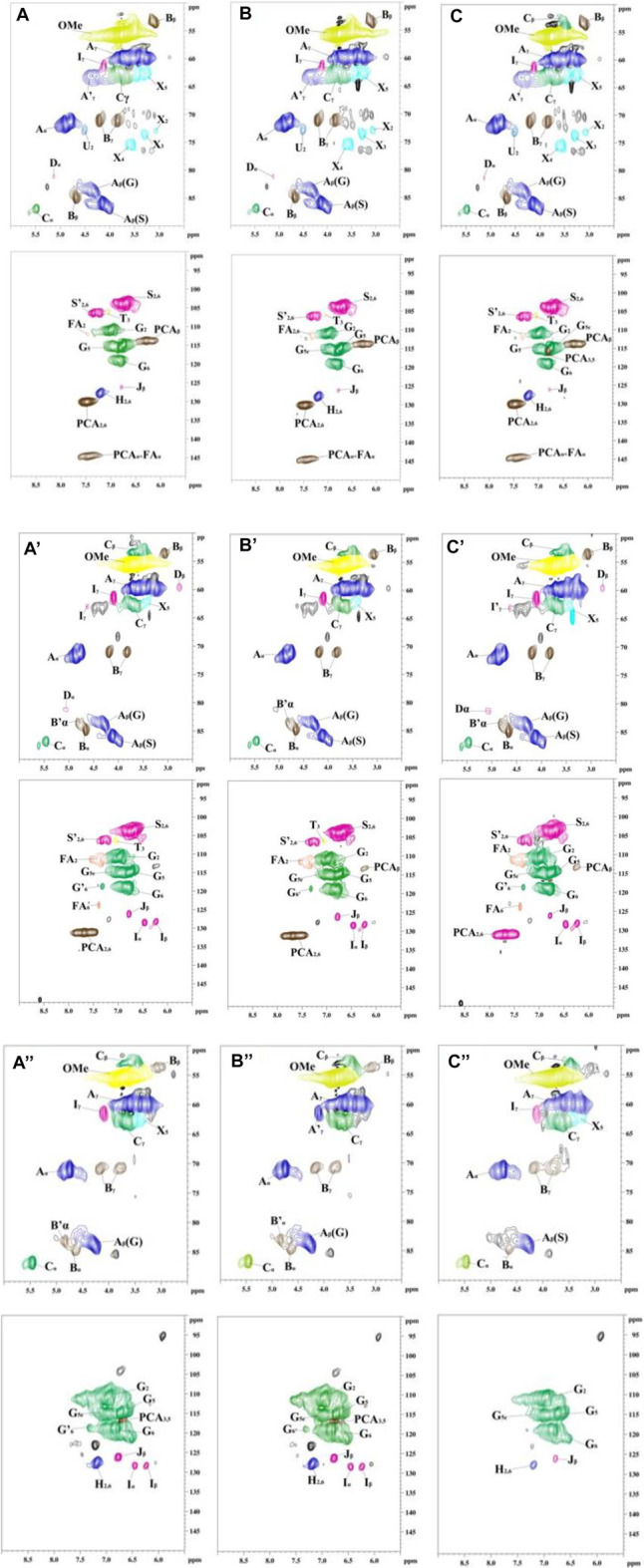
The 2D-HSQC nuclear magnetic resonance (NMR) spectra of all prepared milled wood lignin (MWL) samples from different biomass by different milling times [**(A–C)** are the MWL from bamboo residues with 3-, 5-, and 7-h milling time, respectively; **(A′–C′)** are the MWL from poplar sawdust with 3-, 5-, and 7-h milling time, respectively; **(A"–C")** are the MWL from larch sawdust with 3-, 5-, and 7-h milling time, respectively].

**FIGURE 2:**
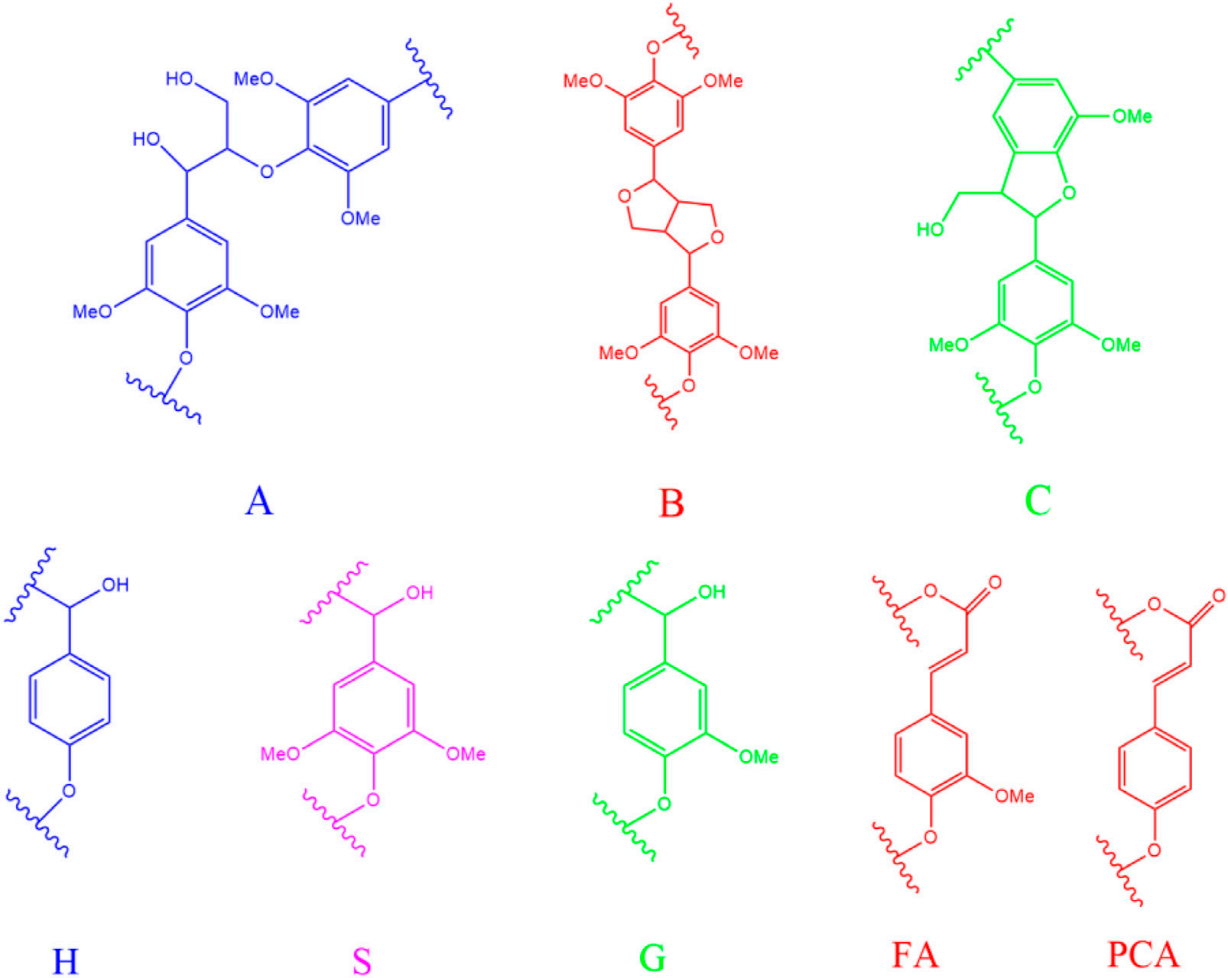
The structure diagram of lignin substructures in the spectra [**(A)**: *ß*-O-4; **(B)**: *ß*-β; **(C)**: *ß*-1; H, p-hydroxyphenyl units; S, syringyl units; G, guaiacyl units; FA, ferulate; PCA, p-coumarate).

To indepthly elucidate the structure features of MWL samples in bamboo residues, poplar sawdust, and larch sawdust by different milling times, 2D-HSQC NMR analysis was carried out by dissolving lignin in DMSO-d_6_. The obtained NMR spectra of all MWL samples and the main structure diagram of lignin substructures in the spectra are shown in Figures 1 and 2, respectively.

In the side-chain (*δ*_C_/*δ*_H_ 90–50/6.0–2.5) spectra, the substructures of *ß*-O-4 (A), *ß*-β (B), and *ß*-5 (C) in all MWL samples can be clearly observed by identifying their corresponding C–H correlation signals (Jiang et al., 2017b; Huang et al., 2020; Wang R. et al., 2021; Wang Z. et al., 2021). In addition, the lignin units of syringyl units (S), guaiacyl units (G), or p-hydroxyphenyl units (H) can also be observed in the aromatic region (*δ*_C_/*δ*_H_ 135–100/8.5–5.5) spectra of MWL samples with some differences (Dong et al., 2020; Wang R. et al., 2021). Specifically, the S, G, and H units can be found in the MWL spectra of bamboo residues, while only S/G units and G/H units existed in the MWL spectra of poplar sawdust and larch sawdust, respectively. Overall, the results indicated that increasing the ball milling time with different times did not show the performance in changing the phenylpropane structure of lignin units in all MWL samples, which is in accordance with the work of Huang et al. (2011).

It should be pointed out that only the overview of the presented lignin substructures in MWL samples can be obtained in Figure 1, which cannot provide the quantitative changes in each substructure. Hence, the amount of lignin substructures in MWL samples from different biomass were semiquantitatively calculated according to their signal clusters in 2D-HSQC spectra. The results were expressed based on 100 aromatic rings (100Ar) and are shown in Table 3. Table 3 shows that a decreased trend was observed for the amount of *ß*-O-4 in MWL samples of different biomass treated by increased ball milling time. Specifically, the amount *ß*-O-4 in MWL of bamboo residues, poplar sawdust, and larch sawdust were decreased from 68.2/100Ar to 60.1/100Ar, from 58.7/100Ar to 55.5/100Ar, and from 66.0/100Ar to 59.4/100Ar, respectively, when the ball milling time was prolonged from 3 to 7 h. In the work of Wang R. et al. (2021), they also found that the increased ball milling time indeed showed a performance in degrading the *ß*-O-4 in the isolated lignin from different biomass, resulting in a decreased amount, while the amount of *ß*-β in MWL samples were increased when prolonging the ball milling time, indicating it is more recalcitrant than *ß*-O-4 to be degraded during ball milling process. From Table 3, it can be obviously found that the *ß*-O-4 was the major linkage in all MWL samples, which is in agreement with the report of Kishimoto et al. (2006) that the frequency of *ß*-O-4 linkage accounts for 40%–65% in the lignin biomacromolecule.

**TABLE 3:** The amount of lignin substructures and S/G ratio of prepared MWL samples from biomass at different milling times.

Biomass	Milling time (h)	Amount of lignin substructures (100Ar)			S/G ratio^a^
		β-O-4 (A)	β-β (B)	β-5 (C)	
Bamboo residues	3	68.2	12.7	4.1	1.0
	5	61.3	15.6	4.8	1.1
	7	60.1	16.2	4.1	1.1
Poplar sawdust	3	58.7	10.7	5.9	0.8
	5	56.4	14.7	8.3	0.8
	7	55.5	25.1	5.4	0.7
Larch sawdust	3	66.0	15.2	7.5	/
	5	60.9	16.6	6.6	/
	7	59.4	16.2	4.2	/

Note. a: S/G = (I_S2,6_/2)/I_G2_.

Generally, the value of S/G ratio is the key index to evaluate which kind of lignin can be easily isolated during ball milling process (Wen et al., 2015; Jiang et al., 2017a). Table 3 shows that there was no significant difference in the S/G ratio for the MWL samples from different milling time for the bamboo residues (∼1.0) and poplar sawdust (∼0.8), while MWL samples from bamboo residues possessed a higher S/G ratio than that of poplar sawdust, indicating that the S-type lignin in poplar sawdust is more recalcitrant to be isolated. In the work of Capanema et al. (2015), they found that the obtained lignin from hardwood with a greater content of S-type unit shows a tendency to endow lignin with a higher amount of *ß*-O-4 linkage. Hence, it can be speculated that the higher value of S/G ratio in MWL samples from bamboo residues might be the reason why they had the highest amount of *ß*-O-4 than that of MWL samples from poplar sawdust. Based on the aforementioned results, it can be seen that increasing ball milling time indeed degraded the *ß*-O-4 in the obtained MWL sample, while it showed little effect on the ratio of lignin unit type in the MWL biomacromolecule.

### Effect of different milling time on the functional group of prepared milled wood lignin samples

Generally, the degradation of substructures (β-O-4, *ß*-β, *ß*-1, et al.) of lignin inevitably affected the content of the functional group of the prepared lignin (Shao et al., 2020). Hence, quantitative ^31^P NMR technology was performed to analyze the changes in the functional group (aliphatic hydroxyl and phenolic hydroxyl) of the prepared MWL samples in bamboo residues, poplar sawdust, and larch sawdust by different milling times. From Table 4, it can be seen that prolonging the milling time resulted in the prepared MWL samples with the increased contents of aliphatic hydroxyl and phenolic hydroxyl groups. The reason for these results can be explained by the increased degradation degree of *ß*-O-4, which can result in the lignin with enhanced amount of hydroxyl groups. In addition, it is found that increasing the ball milling time from 5 to 7 h did not seriously affect the contents of aliphatic hydroxyl and total phenolic hydroxyl groups in all MWL samples. These results were similar to the work of Wang R. et al. (2021) wherein increasing ball milling time showed a small effect on the structure of lignin during isolation process. It should be pointed out that there was no significant difference in the contents of functional groups in MWL samples from different biomasses treating by the same ball milling time.

**TABLE 4:** The contents of functional groups in MWL samples from different biomass (mmol/g).

	Milling time (h)	Aliphatic hydroxyl	Phenolic hydroxyl		Total phenolic hydroxyl	COOH
			Condensed phenolic OH	Noncondensed phenolic OH		
Bamboo residues	3	1.8	1.6	1.5	3.1	0.3
	5	2.1	1.6	2.4	4.0	0.3
	7	2.2	1.8	2.4	4.2	0.5
Poplar sawdust	3	1.8	1.6	2.1	3.7	0.5
	5	2.6	1.5	2.6	4.1	0.6
	7	2.9	1.6	2.6	4.2	0.5
Larch sawdust	3	1.9	1.8	1.8	3.6	0.4
	5	1.9	1.9	2.1	4.0	0.4
	7	2.2	1.9	2.2	4.1	0.5

## Conclusion

In this work, ball milling with different times (3, 5, and 7 h) using ZrO_2_ bowl with ZrO_2_ balls (ΦA = 1 cm) at 600 rpm was carried out to isolate the MWL samples in poplar sawdust, larch sawdust, and bamboo residues. It is found that milling time with 3 and 7 h was sufficient to isolate the representative lignin with yields over 30% for bamboo residues and poplar sawdust, respectively, while more than 7 h should be carried out to isolate the representative lignin in larch sawdust. During the ball milling process with prolonged time, the prepared MWL possessed a smaller substructure of *ß*-O-4, lower molecular weight, and higher content of hydroxyl groups, which was due to the occurred degradation. To prepare the representative MWL in different biomass, not only the ball milling time should be considered; it also should be focused on the degradation degree of the substructure of lignin.

## Data Availability

The original contributions presented in the study are included in the article/Supplementary Material. Further inquiries can be directed to the corresponding author.
